# Subclinical hypothyroidism and cognitive function in people over 60 years: a systematic review and meta-analysis

**DOI:** 10.3389/fnagi.2015.00150

**Published:** 2015-08-11

**Authors:** Abimbola A. Akintola, Steffy W. Jansen, David van Bodegom, Jeroen van der Grond, Rudi G. Westendorp, Anton J. M. de Craen, Diana van Heemst

**Affiliations:** ^1^Department of Gerontology and Geriatrics, Leiden University Medical CenterLeiden, Netherlands; ^2^Leyden Academy on Vitality and AgeingLeiden, Netherlands; ^3^Department of Radiology, Leiden University Medical CenterLeiden, Netherlands; ^4^Department of Public Health, Center for Healthy Ageing, University of CopenhagenCopenhagen, Denmark

**Keywords:** cognition, subclinical hypothyroidism, elderly, meta-analysis, systematic review

## Abstract

Subclinical hypothyroidism (SCH), defined as elevated thyroid stimulating hormone (TSH) and normal thyroid hormone levels, and cognitive impairment are both common in older people. While the relation between overt hypothyroidism and cognitive impairment is well established, data on the association between SCH and cognitive impairment are conflicting. This systematic review and meta-analysis was performed to assess available evidence on the association of SCH with cognition in community dwelling, relatively healthy older adults. PubMed, EMBASE, Web of Science, COCHRANE, CINAHL, PsycINFO, and Academic Search Premier (January 1966 to April 1, 2015) were searched without language restrictions, as were references of key articles, for studies on the association between SCH and cognition in older adults (>60 years). These studies were reviewed by two independent reviewers according to predefined criteria for eligibility and methodological quality, and data were extracted using standardized forms. Of the 844 reports initially identified, 270 remained after exclusion of duplicates. Of the 270, 15 studies comprising 19,944 subjects, of whom 1,199 had subclinical hypothyroidism were included. Data from the 15 studies was pooled, and meta-analyzed cross-sectionally for global cognition [assessed by Mini-Mental State Examination (MMSE)], executive function, and memory, using random effects models. Pooled effect size (ES) for MMSE was −0.01 (95% CI −0.09, 0.08), with heterogeneity (*I*^2^) of 55.1%. Pooled ES was < 0.001 (95% CI −0.10, 0.09) for executive function (*I*^2^ = 13.5%), and 0.01 (95% CI −0.12, 0.14) for memory (*I*^2^ = 46.9%). In addition, prospective analysis including four studies showed pooled ES of 0.033 (95% CI −0.001 − 0.067) for MMSE (*I*^2^ < 0.001%), indicating that subclinical hypothyroidism was not significantly associated with accelerated cognitive decline. This systematic review and meta-analysis provides no evidence that supports an association between SCH and cognitive impairment in relatively healthy older adults.

## Introduction

Overt adult onset hypothyroidism, which is marked by elevated thyroid stimulating hormone (TSH) levels and reduced levels of circulating thyroid hormones, has been associated with increased risk of deficits in specific cognitive domains including attention, concentration, memory, perceptual function, language, executive function, and psychomotor speed (Constant et al., 2005; Davis and Tremont, 2007; Samuels, 2008; Correia et al., 2009). However, controversies persist as to whether subclinical hypothyroidism (SCH), defined as mild elevation of TSH in the presence of normal free thyroxine (fT4), is associated with declines in these specific cognitive domains. This is especially relevant in the older adults, as the prevalence of subclinical hypothyroidism increases with age and is estimated to be up to 22% in women aged more than 60 years and somewhat lower in men (Sawin et al., 1985; Canaris et al., 2000).

Many studies have investigated whether subclinical hypothyroidism is associated with increased risk of cognitive impairment (Manciet et al., 1995; Cook et al., 2002; Gussekloo et al., 2004; Roberts et al., 2006; Cardenas-Ibarra et al., 2008; Hogervorst et al., 2008; Ceresini et al., 2009; John et al., 2009; Park et al., 2010; de Jongh et al., 2011; Resta et al., 2012; Yamamoto et al., 2012; Wijsman et al., 2013; Formiga et al., 2014; Parsaik et al., 2014). However, the data are conflicting, and epidemiological studies that investigated this relationship have reported inconsistent findings. Furthermore, due to use of different TSH cut-off values, methodological differences, application of varying cognitive tests for different cognitive domains, and diverse reporting of results by these studies, the interpretability and comparability of their findings are hindered.

Here, we performed a systematic review of available evidence from both cross-sectional and prospective studies on the association between subclinical hypothyroidism and cognition in the older adults. Furthermore, we performed a meta-analysis to quantify the magnitude of the associations between subclinical hypothyroidism and both global cognition as well as two specific cognitive domains, namely executive function and memory.

## Methods

### Data sources and search strategy

A systematic literature search was conducted of articles published from January 1966 to April 1, 2015 on the association between subclinical hypothyroidism and cognition in the elderly. PubMed, EMBASE, Web of Science, COCHRANE, CINAHL, PsycINFO, and Academic Search Premier were searched (Datasheet 1). The design of the electronic search strategy was done in consultation with an expert information specialist. A thorough search was conducted on the bibliographies of key articles in the field and these were included in this review. To avoid missing any relevant study in the search, broadly defined terms were used (Datasheet 1). Reference lists of key articles were also searched for relevant articles that could have been missed.

### Study selection

Two independent reviewers (AAA and SWJ) screened the extracted citations for eligibility. To maximize the quality and comparability of the studies, general inclusion and exclusion criteria were defined *a priori* (Table 1). The titles, abstracts and later the full-texts of the search results were screened—the studies included were those that assessed the cognitive status of relatively healthy (community dwelling, and considered healthy by the authors of the original articles) elderly (aged 60 years and above) participants with subclinical hypothyroidism.

**Table 1:** **Selection criteria for eligibility for inclusion or exclusion**.

**Inclusion criteria**	**Exclusion criteria**
Human studies	Animal studies
Median/mean age 60 or above	Younger than 60
Subclinical hypothyroidism (SCH) defined as: -Elevated TSH and normal fT4; -All self-defined subclinical hypothyroidism Elevated serum TSH in association with normal total or free T4- and T3-values High-normal TSH and abnormal response to TRH Elevated serum TSH with normal thyroid hormone levels, without symptoms that could be explained by overt hypothyroidism	SCH not defined
Relatively healthy elderly participants Healthy as determined by the authors of the original articles	Full blown depression, psychiatric symptoms, neurological disorders as Parkinson's disease or predefined dementia, substance abuse
Free living/community dwelling	Hospitalized patients
Original research articles including prospective studies, randomized-controlled trials, etc. that provide baseline data	Systematic reviews, meta-analyses, reviews, conference abstracts, web pages
Cognitive measure and domain specified	Cognitive domain not well defined, e.g. “mood,” “quality of life,” “mental health” etc.
All languages	
	Duplicates

Subclinical hypothyroidism is defined as elevated TSH and normal fT4 (Helfand, 2004). However, controversies exist as to the upper limit of the TSH reference range. Several reviews suggest a TSH upper limit of 4.5–5.0 mIU/L (Helfand, 2004; Surks et al., 2004), but some authors suggest that the upper limit of the TSH range should be reduced to 2.5–3.0 mIU/L, based on a higher risk of progression to overt hypothyroidism and a higher prevalence of anti-thyroid antibodies than in euthyroid participants (Vanderpump et al., 1995). In the absence of a consensus, the use of a specific TSH upper limit was not pre-specified in this systematic review to define subclinical hypothyroidism. Furthermore, fT4-values were considered normal if indicated as normal by the authors, even if data on fT4 were not presented.

Studies done on participants with depression [according to the Diagnostic and Statistical Manual of Mental Disorders (DSM) criteria], dementia, psychiatric symptoms, neurological disorders e.g. Parkinson's disease, and other chronic systemic illnesses were excluded. Furthermore, participants using thyroid medications were excluded. Three relatively large studies that measured health status of participants with an elevated TSH were initially included. However, they were later excluded because assessment of mood, and general and mental health status was done qualitatively, without specifying whether global cognition or specific cognitive domains were measured (Razvi et al., 2005; Gulseren et al., 2006; Vigario et al., 2009).

### Data extraction and quality assessment

From each study that met the eligibility criteria, information was extracted about study design (prospective or cross-sectional), participant characteristics, criteria used to define subclinical hypothyroidism, cognitive tests applied and domains tested, and study results (effect estimates, variables included for adjustments, or matching procedures) using a standardized data-collection form.

The two reviewers (AAA and SWJ) independently assessed the methodological quality of included studies using a pre-defined list of criteria (Egger et al., 1997; Stroup et al., 2000) (Datasheet 2). In total, 11 key indicators were used to systematically assess study quality. These were (1) clarity of hypothesis, (2) population studied (convenience sample vs. population-based, defined as a random sample of the general population), (3) clear definition of subclinical hypothyroidism (indication of TSH cut-off and fT4-values that were used in the study), (4) detailed description of study materials and methods, (5) validity of measurements and cognitive tests, (6) number of cognitive domains tested (global cognition, executive function, and/or memory), (7) clear description of statistical methods, (8) adjustments/correction for potential confounders, (9) clear presentation of results, (10) generalizability to other populations, and (11) method of outcome adjudication [use of formal adjudication procedures, defined as having clear criteria for the outcome (cognitive impairment)]. A score of “0” (lacking), “1”(incomplete), or “2” (complete) was assigned to each of the key indicators per study, with a maximum total score of 22.

### Data synthesis and statistical analysis

Authors were contacted when necessary to request more detailed data on the association between subclinical hypothyroidism and cognition in older adults (Gussekloo et al., 2004; de Jongh et al., 2011; Formiga et al., 2014). The most adjusted estimates and SD/SE were used for analysis, where available. In instances where participants were divided into groups based on TSH-values (tertiles/quartiles), the mean TSH-value for the whole group was used.

Data was qualitatively synthesized and assessed for the number of participants that were included, the definition of subclinical hypothyroidism applied, the cognitive tests that were used and the cognitive domains that were measured. Meta-analysis was done by comparing estimates from participants with subclinical hypothyroidism with those from euthyroid participants, using data from both cross-sectional studies and baseline data from prospective studies for the cross-sectional analysis. Thus, only studies that provided these estimates were included in the meta-analysis. Using Hedges method (Hedges and Vevea, 1998), pooled estimates with standard error were calculated first from cross-sectional analysis of available studies, and then for the prospective data, using the same approach.

To make effect estimates comparable between studies, effect sizes (ES) were calculated from calculated means with standard deviation of participants with subclinical hypothyroidism compared to euthyroid participants. For studies that used >1 cognitive test (Cook et al., 2002; Gussekloo et al., 2004; John et al., 2009; Park et al., 2010; Wijsman et al., 2013; Parsaik et al., 2014), a pooled ES was calculated for each study. After calculating an ES for each study, a meta-analysis was performed using a random effects model. The random effects model was applied, because it takes into account the heterogeneity between the studies. All statistical analyses were performed using STATA version 10. Cochrane *Q*-test and *I*^2^ index with a conservative *p*-value of 0.10 were used to evaluate the heterogeneity across individual studies. *I*^2^-values of < 25% were considered reflective of low, between 25 and 50% of moderate and >50% of high heterogeneity between studies.

## Results

### Study selection

**Figure 1:**
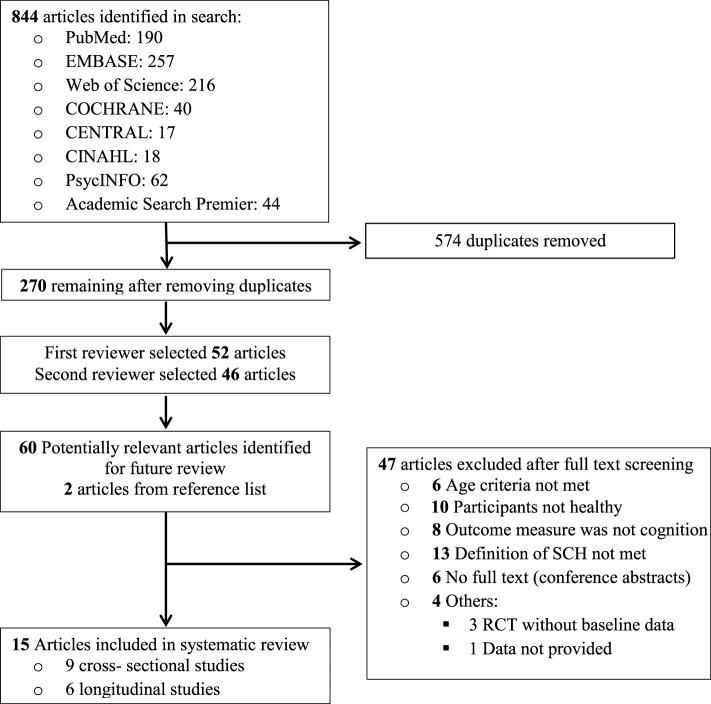
**Flowchart showing the literature search for the systematic review**.

Of the 844 reports initially identified, 270 remained after exclusion of duplicates. Of the 270 reports, 210 were excluded that were unrelated to the association between subclinical hypothyroidism and cognition in the elderly (Figure 1), leaving 60 articles for full text analysis. Two more articles were selected from reference lists of relevant articles. Of the 62 articles that were selected for detailed (full text) evaluation, full texts were not available for six studies. Additionally, 10 studies were excluded because the participants were not considered healthy, another 13 because the definition of subclinical hypothyroidism (high TSH and normal fT4) was not met and four because data was not available for systematic review [three were randomized controlled trials (RCTs) without baseline data, and one study reported qualitative results]. Furthermore, eight studies that did not measure cognition as endpoint were excluded—these either measured mental health by means of questionnaires, or studied depression. Six other studies were excluded because the mean/median age of the participants was less than 60 years (Figure 1).

Two studies each were reported in Spanish and Dutch, respectively, one in Italian and another in Czech. The researchers (AAA and SWJ) themselves translated the two Dutch articles into English. Another article was translated from Czech to English by the author himself (Jensovsky et al., 2000). The other foreign language articles were translated to English by the researchers' colleagues that spoke the language. These articles were all later excluded after full-text analysis. When similar data were published more than once (Gussekloo et al., 2004, 2006; Wiersinga, 2006), the article with the most definitive and extractable data was included (Gussekloo et al., 2004). One study was later dropped because it studied the effect of subclinical hypothyroidism on demented and non-demented elderly using only clinical dementia rating, thus was incomparable with the other selected studies in terms of results (Ganguli et al., 1996). Fifteen observational studies met the eligibility criteria.

### Study characteristics

Table 2 shows the characteristics of the nine cross-sectional and six prospective studies that were included in the review. In total, the 15 studies comprised 19,944 participants, of whom 1,199 had subclinical hypothyroidism. The upper reference limit of TSH (TSH cut-off) to define subclinical hypothyroidism ranged from 3.6 to 10 mIU/L. A total of 13 out of the 15 studies also reported fT4 measurement. The studies used varying cognitive tests to measure a wide range of cognitive domains. The cognitive domains that were covered included global cognition, executive function, memory, general intelligence, attention, and concentration, visio-spatial organization, language, and cognitive or psychomotor speed. These cognitive domains were merged into three main domains, namely global cognition, executive function, and memory, as shown in Table 3. The cognitive tests that were used for each of these cognitive domains are also presented in Table 3.

**Table 2:** **Characteristics of studies included in the systematic review on the association between subclinical hypothyroidism (SCH) and cognitive impairment in older adults**.

	**First author**	**Type of study**	**Study (Follow-up in years)**	***N* Total**	***N* with SCH**	**Mean age (Years)**	**Cut off TSH (ref. range) mIU/l**	**fT4 pmol/l**	**Cognition tests**	**Study quality**
1	Roberts et al., 2006	Cross-sectional	N/A^*^	5865	168	73.6	>5.5	9.0–20.0	MMSE, MEAMS	21
2	Wijsman et al., 2013	Prospective	Prosper study (3 years)	5.154	161	75	>4.5 (0.45–4.5)	12–22	MMSE, Stroop, LDCT, WLT. (Immediate and delayed)	22
3	Parsaik et al., 2014	Cross-sectional	N/A^*^	1904	141	81	>10	12.87–12.04	WAIS-R: TMT, DSST; BNT, CFT, PCBD, WMS (logical memory I and visual reproduction II)	21
4	Park et al., 2010	Cross-sectional	N/A^*^	918	164	76	>4.1 (0.4–4.1)	9.0–23.2	MMSE, DS, FAB, CERAD-K-N including CFT, BNT-modified, CPT, WLMT, WLRT, WRLRcT, CRT	19
5	de Jongh et al., 2011	Prospective	Longitudinal aging study, Amsterdam,(10.7 years)	1219	64	75.5	>4.5 (0.3–4.5)	11–22	MMSE, RPM, and the coding task	20
6	Hogervorst et al., 2008	Prospective	MRC cognitive function and aging study (2 years)	1047	33	73.6	>4.8	13–23	MMSE, WMS-revised	20
7	Gussekloo et al., 2004	Prospective	Leiden 85 + study (3.7 years)	558	30	85	>4.8	13–23	MMSE, Stroop, LDCT, WLT (immediate and delayed).	21^#^
8	John et al., 2009	Cross-sectional	N/A^*^	489	286	>60	>10.0 (0.3–10.0)	Not indicated	SILS, TMT-b, SDMT, JLO, BD and LNS from WAIS, AN, BNT-modified, CVLT, EBMT, Faces I and II from WMS	17
9	Resta et al., 2012	Cross-sectional	N/A^*^	391	42	74.3	>3.6 (8.0–17.0 pg/mL)	8.1–15.43	MMSE, PMT, and MT	16
10	Ceresini et al., 2009	Cross-sectional	N/A^*^	1117	25	77	>4.7	9.9–28.2	MMSE	18
11	Formiga et al., 2014	Prospective	OCTABAIX study (3 years)	328	20	85	>5	10–26	MMSE (MEC, Spanish version)	19^#^
12	Manciet et al., 1995	Cross-sectional	N/A^*^	425	26	74.4	>4.5 (0.5–4.5)	16–29	MMSE, WAIS, BVRT, ZBT, IT	16
13	Yamamoto et al., 2012	Prospective	Japanese study (1 year)	229	15	80.9	Not indicated	Not indicated	MMSE, revised hasegawa dementia scale	16
14	Cook et al., 2002	Cross-sectional	N/A^*^	97	15	74	4.0 (0.4–4)	Not indicated	MMSE, AVLT, DSCT from WAIS, *N* Back test, backward DS	16
15	Cardenas-Ibarra et al., 2008	Cross-sectional	N/A^*^	253	9	80	>4.5	Not indicated	MMSE	12

AN, Animal naming; AVLT, Auditory verbal learning test; BD, block design; BNT, Boston naming test; CFT, category fluency test; CPT, constructional praxis test; CRT, constructional recall test; CVLT, California Verbal Learning Test; CVMT, continuous visual memory test; DS, digit spans forward and backward of WAIS-R; DSCT, Digit symbol coding test (from WAIS); DSST, Digit symbol substation test; EBMT, East Boston Memory Test; FAB, Frontal assessment battery; IT, Isaacs set test of verbal fluency; JLO, Judgment of line orientation; LDCT, letter digit coding test; LNS, Letter-number sequencing; LW, list of words; MEAMS, Middlesex elderly assessment of mental state (12 scores); MEC, Mini mental state examination (35 scores); MMSE, Mini-Mental State Examination(30 scores); MMMSE, Modified Mini-Mental State Examination; MT, Matrix test; PMT, Prose memory test; PCBD, Picture completion and block design; RPM, raven progressive matrices test; RW, Rey's words immediate and delayed recall; SDMT, symbol digit modalities test; SILS, Shipley Institute of Living scale; Stroop, Stroop color word test; TMTA&B, trail making test A and B; WAIS, Wechsler adult intelligence scale; WAIS-R, Wechsler adult intelligence scale-revised; WFT, word fluency test; WLMT, word list memory test; WLRT, Word list recall test; WRLRcT, Word list recognition test; WLT, word learning task; WMS, Wechsler memory scale; ZBT, Zazzos barring test.

* N/A: Not applicable.

# Score based on published and unpublished data provided by the author.

**Table 3:** **Cognitive tests and domains used for meta-analysis**.

**Cognitive domain**	**Measures and cognitive tests**
Global cognition	MEAMS, MMSE, MMMSE, 3MSE
Memory (including tests for language)	AN, AVLT, CRT, CVMT, DS, EBMT, FMT, IPALT, LDCT, LW, *N*-back test, PMT, PWLT, RCFT, SRT, WLT, RBP, RW, WLMT, WLRT, WMS, WRLRcT, Language: AN, BNT, CFT, CVLT, COWAT, IT, OR, RW, WFT, WD, ZBT
Executive function	BD, FAB, DSST, GNG, LMN, MT, PM, RPM, SILS, TMT, WAIS, WFT, Attention and concentration: CST, DS, LNS, PASAT, SDMT, Stroop, TMTA&B Visuo-spatial organization: CC, CoS, CPT, FR, JLO, HT, PCBD, ScT, SDMT, TMT(Part A), WAIS-R Cognitive or psychomotor speed: DSCT, WAIS-R, TMT(Part A), WFT

AN, animal naming; AVLT, Auditory verbal learning test; BD, block design; BNT, Boston naming test; CFT, category verbal fluency test; COWAT, Controlled oral word; CPT, constructional praxis test; CRT, constructional recall test.; CST, concept shifting test; CVLT, California Verbal Learning Test; CVMT, continuous visual memory test; DS, digit spans forward and backward of WAIS-R; DSCT, Digit symbol coding test (from WAIS); DSST, Digit symbol substitution test; FMT, Milner facial memory test; EBMT, East Boston Memory Test; FAB, Frontal assessment battery; FR, figure rotation from the Schaie-Thurstone adult mental abilities test; GNG, Go-No-Go; HT, Hooper test; IPALT, Inglis paired associates learning test; IT, Isaacs set test of verbal fluency; JLO, Judgment of line orientation; LDCT, letter digit coding test; LMN, Luria m's and n's; LNS, Letter-number sequencing; LW, list of words; 3MSE, Modified MMSE (100 scores); MEAMS, Middlesex elderly assessment of mental state (12 scores); MMSE, Mini mental state examination (30 scores); MMMSE, Modified Mini-Mental State Examination; MT, Matrix test; OR, oral reading; PASAT, paced auditory serial addition task; PM, Porteus maze; PMT, Prose memory test; PCBD, Picture completion and block design; PWLT, picture word learning test; RBP, Rivermead behavioral profile; RCFT, Rey-Osterrieth complex figure test; RPM, raven progressive matrices test; RW, Rey's words immediate and delayed recall; ScT, scribble test; SDMT, symbol digit modalities test; SILS, Shipley Institute of Living scale; SRT, selective reminding test (Buschke); TMTA&B, trail making test A and B; WAIS, Wechsler adult intelligence scale; WAIS-R, Wechsler adult intelligence scale-revised; WD, word discrimination; WFT, word fluency test; WLMT, word list memory test; WLRT, Word list recall test; WRLRcT, Word list recognition test; WLT, word learning task; WMS, Wechsler memory scale; ZBT, Zazzos barring test.

### Systematic review

In total, 1,199 participants with subclinical hypothyroidism were included in the systematic review. From the 15 studies in our systematic review, 12 studies indicated a lack of significant association between subclinical hypothyroidism and cognitive impairment in the elderly. These studies comprised 1,109 participants with subclinical hypothyroidism and therefore contributed 92.5% of the population sampled to the outcome of the systematic review. Of the remaining three studies, two found an association, and one was inconclusive (Hogervorst et al., 2008). The inconclusive study demonstrated an association between log transformed TSH levels with decreasing MMSE performance in hypothyroid participants, but it was not specified whether the observed association was with overt hypothyroidism or with subclinical hypothyroidism. This study was included in the systematic review but excluded from meta-analysis.

A total of two studies found an association between subclinical hypothyroidism and cognition in the elderly. The first study found (in 15 participants with subclinical hypothyroidism) that high TSH levels were associated with worse verbal memory and MMSE scores but not with speed of performance (Cook et al., 2002). The second study found (in 42 participants with subclinical hypothyroidism) that performances in MMSE and Prose memory test were lower in participants with subclinical hypothyroidism compared to euthyroid participants (Resta et al., 2012). Performance in matrix test was also slightly lower in subclinical hypothyroidism, but this was not significant. Summarily, from the studies that observed a significant association between subclinical hypothyroidism and cognitive impairment, the cognitive domains affected were global cognition as assessed via MMSE; executive function as assessed via matrix test; and memory as assessed via auditory verbal learning test, prose memory test, and verbal fluency. The two studies combined comprised 57 participants with subclinical hypothyroidism and contributed only 4.75% to the overall population with subclinical hypothyroidism and to the outcome of the systematic review.

### Meta-analysis

**Figure 2:**
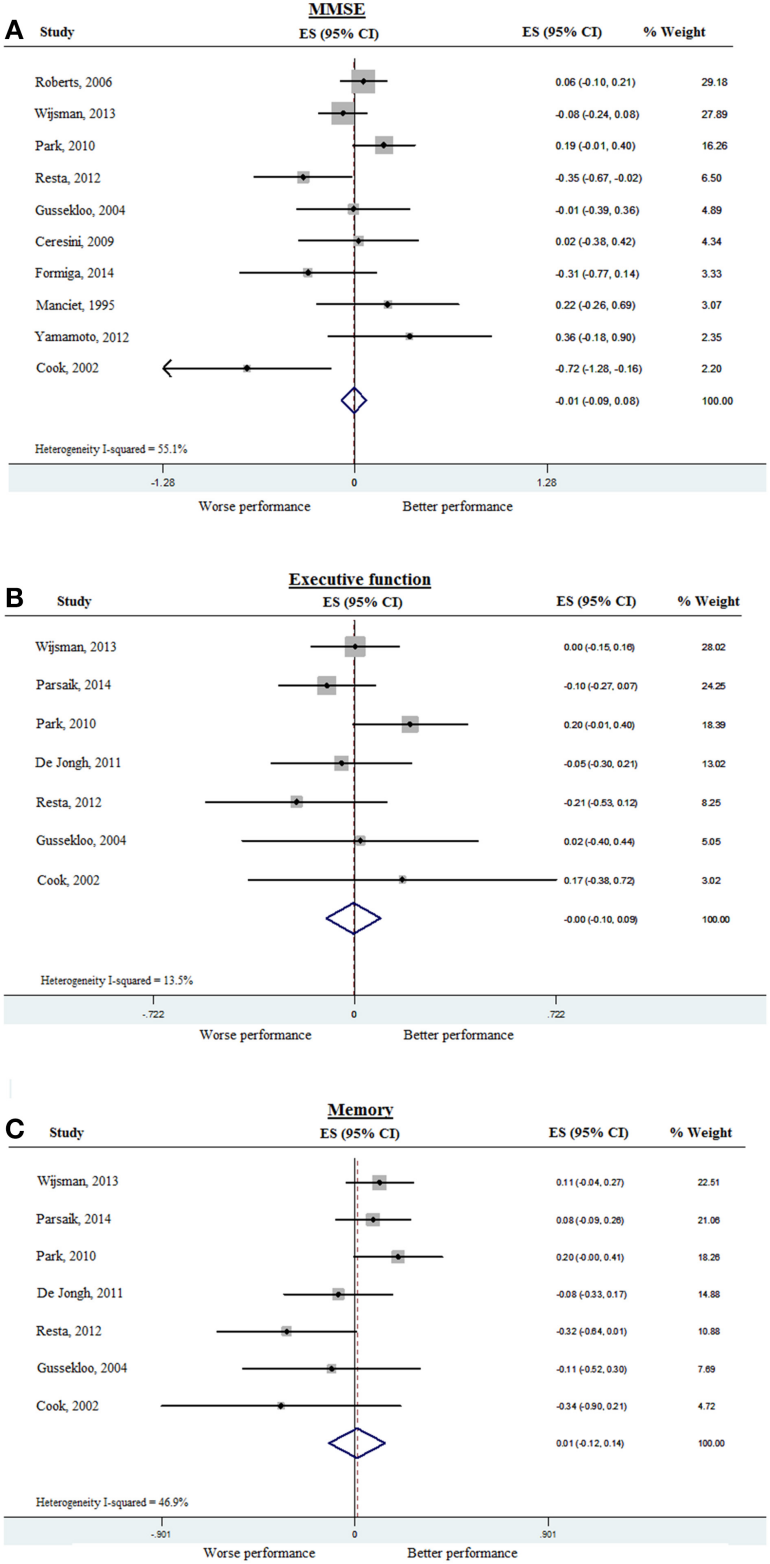
**Forest plots depicting the cross-sectional associations observed between subclinical hypothyroidism (compared to controls) and cognitive performance in 10 studies, arranged according to the weight of the studies**. Data was pooled from cross-sectional studies and baseline data of prospective studies. **(A)** Association between subclinical hypothyroidism and global cognition as measured by MMSE, **(B)** Association between subclinical hypothyroidism and executive function, and **(C)** Association between subclinical hypothyroidism and memory. The pooled effect sizes are displayed as diamonds. MMSE, Mini-mental state examination.

To assess whether subclinical hypothyroidism was associated with impairment of various cognitive domains, we analyzed MMSE separately as a measure of global cognition. Ten out of the 15 studies provided MMSE results either at baseline or at follow-up. The rest of the cognitive tests were categorized into tests of executive function or of memory, as shown in Table 3. Data from the 15 studies was pooled first for cross-sectional analysis, and meta-analyzed separately for global cognition (MMSE), executive function, and memory. The pooled ES for MMSE was −0.01 (95% CI −0.09, 0.08), with heterogeneity (*I*)^2^ of 55.1% (Figure 2A). Pooled ES was < 0.001 (95% CI −0.10, 0.09) for executive function (*I*^2^ = 13.5%) (Figure 2B), and 0.01 (95% CI −0.12, 0.14) for memory (*I*^2^ = 46.9%) (Figure 2C). These analyses indicated that available evidence does not support an association of subclinical hypothyroidism with worse performance in MMSE, executive function or global cognition.

**Figure 3:**
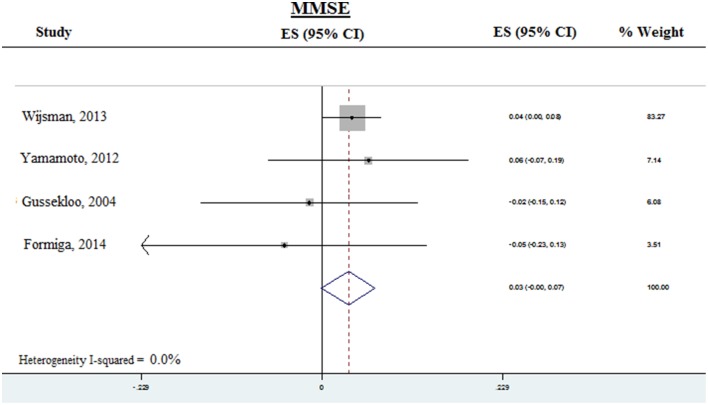
**Forest plots depicting the prospective analysis of associations observed between subclinical hypothyroidism and decline in global cognition as measured by MMSE, arranged according to the weight of the studies**. The pooled effect sizes are displayed as diamonds. MMSE, Mini-mental state examination.

Prospective analysis was done for MMSE in four studies from which prospective data was available (Figure 3). The pooled ES was 0.03 (95% CI −0.001–0.07) *P* = 0.055, with heterogeneity (*I*^2^) of < 0.001%. Thus, subclinical hypothyroidism was not significantly associated with accelerated decline of global cognition, as assessed by MMSE. Due to the small number of available studies, prospective analysis was not done for executive function or memory.

### Subgroup and sensitivity analyses

Subgroup analyses were performed on studies with similar TSH cut-off values, and in studies with similar study design (cross-sectional or prospective). The ES of these different subgroups were essentially similar, indicating that in this meta-analysis, subclinical hypothyroidism was not significantly associated with cognitive impairment.

## Discussion

On the basis of the findings of this systematic review and meta-analysis we did not find evidence supporting an association of subclinical hypothyroidism with cognitive impairment in relatively healthy, community-dwelling elderly. Out of 15 observational studies, only two small cross-sectional studies (Cook et al., 2002; Resta et al., 2012) observed statistically significant associations between subclinical hypothyroidism and cognitive impairment, namely global cognition (MMSE), and memory. All other studies indicated a lack of association. No evidence was found that the lack of association between subclinical hypothyroidism and cognitive impairment was limited to unadjusted studies, or to studies of lower methodological quality. Meta-analysis of studies from which data for cross-sectional analysis could be retrieved, revealed lack of association between subclinical hypothyroidism and global cognition (assessed by MMSE) as well as lack of association of subclinical hypothyroidism with memory and executive function. Subgroup analyses by type of study design showed a similar trend in the prospective cohort studies group compared with the cross-sectional studies. We also did not find evidence supporting an association of subclinical hypothyroidism with cognitive impairment in a prospective analysis. However, the number of studies retrieved for prospective analysis was low and the study quality (assessed by scoring based on key indicators) varied.

Our results are in line with previous focused reviews (Parle et al., 2010; Joffe et al., 2013) supporting a lack of association between subclinical hypothyroidism and cognitive impairment that largely drew upon the results from large population based studies (Roberts et al., 2006). In contrast, another review conducted on the association between TSH and cognitive impairment in community dwelling and hospitalized elderly (Annerbo and Lökk, 2013) reported some evidence supporting the association between subclinical hypothyroidism and cognitive impairment, which was driven by studies showing an association between thyroid hormones and dementia. Thus, previous observational studies on the association of cognitive impairment and subclinical hypothyroidism have yielded conflicting results. Our finding of lack of association between subclinical hypothyroidism and cognitive impairment is also in line with the results of two placebo controlled randomized clinical trials (Jorde et al., 2006; Parle et al., 2010) that showed no effect of treatment with T4 on cognitive endpoints in participants with subclinical hypothyroidism.

To our knowledge, this is the first meta-analysis to examine the cross-sectional and prospective associations between subclinical hypothyroidism and cognitive impairment using available evidence. By pooling the data from the 15 studies, a total of 19,944 participants, of whom 1,199 had subclinical hypothyroidism were analyzed. This increased the power to detect potential associations and reduced the probability of false-negative results (Resta et al., 2012). Case-control and cross-sectional studies are more susceptible to bias, particularly selection bias for case-control studies (Hulley et al., 2001). Although bias cannot be excluded, almost all the cross-sectional studies that fulfilled our quality criteria demonstrated the absence of a statistically significant association between subclinical hypothyroidism and cognitive impairment (Hogervorst et al., 2008; Park et al., 2010).

Overt hypothyroidism has been associated with global cognitive impairment as well as with impairments in various cognitive domains, notably in memory and executive function. Because thyroid dysfunction can be seen as a continuum, it has been hypothesized that subclinical hypothyroidism might also be associated with mild cognitive impairment. The inverse physiological relationship between circulatory levels of TSH and thyroid hormones implies that in subclinical hypothyroidism, thyroid hormone action may be slightly reduced (even though circulatory thyroid hormones are still in the normal range), which might be associated with subtle defects in specific cognitive domains, including memory and executive function. Moreover, one might speculate that potential associations between subclinical hypothyroidism and cognitive impairment are stronger when TSH is markedly increased (TSH > 10 mIU/L) as compared to mild or moderate increases. Similarly, it was found previously that associations between subclinical hypothyroidism and risk for coronary heart disease and mortality were strongest with a TSH concentration of 10 mIU/L or greater (Rodondi et al., 2010).

This analysis has four main limitations. Firstly, all data were obtained from observational studies, many of which are cross-sectional studies. There is a possibility of bias in the selection of included studies, bias and quality problems in the original studies, publication bias, heterogeneity, and confounding (Stroup et al., 2000). To limit bias in the selection of included studies, broad inclusion criteria were used for studies that provided quantitative data on the risk of cognitive impairment in elderly participants with subclinical hypothyroidism. Furthermore, sensitivity analyses were performed according to differences between the studies and methodological study quality, as recommended (Berlin, 1995; Stroup et al., 2000). Many of the original studies did not have statistically significant results, thus a meta-analysis was conducted to increase the power to find an association. Still, the negative conclusion of this systematic review and meta-analysis may be limited by inherent biases and differences in study designs (Huston and Naylor, 1996). However, the sensitivity analyses performed did not suggest that the presented results meaningfully depended on differences in study designs or other study characteristics.

Secondly, the possibility of misclassification of subjects as having subclinical hypothyroidism cannot be ruled out (Huston and Naylor, 1996). In most of the studies, the diagnosis of subclinical hypothyroidism was based on single assessment of TSH, without repeated confirmatory TSH measurement. This could have resulted in inclusion of individuals with only transiently elevated TSH levels. Furthermore, none of the included studies used age-adjusted TSH reference ranges to enroll the subjects. Since increased age has been associated with an increase in the upper limit of the TSH reference range (Zhu et al., 2006), the use of unadjusted reference ranges may have resulted in misclassification of some elderly participants as having subclinical hypothyroidism. This misclassification may have resulted in underestimation of the association between subclinical hypothyroidism and cognition. However, since the 95% CI around the estimates are quite narrow and the misclassification is likely to be small, a large effect of subclinical hypothyroidism on cognition can be confidently ruled out.

Thirdly, the definitions of subclinical hypothyroidism and cognitive decline were slightly different between the studies. The use of different TSH cut-offs reflects the absence of consensus to define subclinical hypothyroidism (Helfand, 2004; Surks et al., 2004). Some studies used a TSH upper limit of < 4.5 mIU/L (Cook et al., 2002; Park et al., 2010), and the inclusion of those participants may have blunted the effect of any observed associations, since they may not have had subclinical hypothyroidism (Surks et al., 2004). However, the sensitivity analyses pooling more homogeneous studies gave similar results indicating a lack of evidence supporting an association of subclinical hypothyroidism with cognitive impairment. However, one might speculate that potential associations between subclinical hypothyroidism and cognitive impairment might only be present when TSH is markedly increased (TSH > 10 mIU/L). Future studies using individual participant data should be directed at analyzing available evidence for an association between subclinical hypothyroidism and cognition based on TSH categories, as was done previously for associations between subclinical hypothyroidism and coronary heart disease (Rodondi et al., 2010).

Fourthly, there were several differences in methodologies and choice of cognitive domains that were tested in the studies in this systematic review and meta-analysis. Thus, we cannot exclude the possibility that subclinical hypothyroidism might be associated with subtle defects in specific domains that can only be identified using highly specific cognitive tests and measures. Indeed, functional neuro-imaging studies in participants with subclinical hypothyroidism and markedly elevated TSH levels has revealed impairments in working memory and brain areas associated with executive function that reversed by treatment with T4 (Zhu et al., 2006). However, the clinical relevance of such specific measures remains unclear. Moreover, different laboratory methods were used for the measurements of TSH and fT4. In addition, TSH has a distinct circadian rhythm and time of the measurements of TSH was not reported in the articles, which may have affected the results.

In conclusion, this systematic review and meta-analysis provides no evidence that supports an association between subclinical hypothyroidism and cognitive impairment in relatively healthy, community dwelling elderly. However, available prospective studies were limited. Thus, additional large, high-quality studies are needed that will allow for more extended analyses.

### Conflict of interest statement

The authors declare that the research was conducted in the absence of any commercial or financial relationships that could be construed as a potential conflict of interest.

## References

[B1] Annerbo S. Lökk J. (2013). A clinical review of the association of thyroid stimulating hormone and cognitive impairment. ISRN Endocrinol. 2013:856017. 10.1155/2013/856017 24171118 PMC3793467

[B2] Berlin J. A. (1995). Invited commentary: benefits of heterogeneity in meta-analysis of data from epidemiologic studies. Am. J. Epidemiol. 142, 383–387. 7625402 10.1093/oxfordjournals.aje.a117645

[B3] Canaris G. J. Manowitz N. R. Mayor G. Ridgway E. C. (2000). The Colorado thyroid disease prevalence study. Arch. Intern. Med. 160, 526–534. 10.1001/archinte.160.4.526 10695693

[B4] Cárdenas-Ibarra L. Soláno-Velazquez J. A. Salinas-Martínez R. Aspera-Ledezma T. D. Sifuentes-Martinez M. R. Villarreal-Pérez J. Z. (2008). Cross-sectional observations of thyroid function in geriatric Mexican outpatients with and without dementia. Arch. Gerontol. Geriatr. 46, 173–180. 10.1016/j.archger.2007.03.009 17512618

[B5] Ceresini G. Lauretani F. Maggio M. Ceda G. P. Morganti S. Usberti E.. (2009). Thyroid function abnormalities and cognitive impairment in elderly people: results of the Invecchiare in Chianti study. J. Am. Geriatr. Soc. 57, 89–93. 10.1111/j.1532-5415.2008.02080.x 19054181 PMC2631617

[B6] Constant E. L. Adam S. Seron X. Bruyer R. Seghers A. Daumerie C. (2005). Anxiety and depression, attention, and executive functions in hypothyroidism. J. Int. Neuropsychol. Soc. 11, 535–544. 10.1017/S1355617705050642 16212680

[B7] Cook S. E. Nebes R. D. Halligan E. M. Burmeister L. A. Saxton J. A. Ganguli M. (2002). Memory impairment in elderly individuals with a mildly elevated serum TSH: the role of processing resources, depression and cerebrovascular disease. Aging Neuropsychol. Cogn. 9, 175–183. 10.1076/anec.9.3.175.9610

[B8] Correia N. Mullally S. Cooke G. Tun T. K. Phelan N. Feeney J.. (2009). Evidence for a specific defect in hippocampal memory in overt and subclinical hypothyroidism. J. Clin. Endocrinol. Metab. 94, 3789–3797. 10.1210/jc.2008-2702 19584178

[B9] Davis J. D. Tremont G. (2007). Neuropsychiatric aspects of hypothyroidism and treatment reversibility. Minerva Endocrinol. 32, 49–65. 17353866

[B10] de Jongh R. T. Lips P. van Schoor N. M. Rijs K. J. Deeg D. J. Comijs H. C.. (2011). Endogenous subclinical thyroid disorders, physical and cognitive function, depression, and mortality in older individuals. Eur. J. Endocrinol. 165, 545–554. 10.1530/EJE-11-0430 21768248

[B11] Egger M. Davey Smith G. Schneider M. Minder C. (1997). Bias in meta-analysis detected by a simple, graphical test. BMJ 315, 629–634. 10.1136/bmj.315.7109.629 9310563 PMC2127453

[B12] Formiga F. Ferrer A. Padros G. Contra A. Corbella X. Pujol R. (2014). Thyroid status and functional and cognitive status at baseline and survival after 3 years of follow-up: the OCTABAIX study. Eur. J. Endocrinol. 170, 69–75. 10.1530/EJE-13-0722 24144964

[B13] Ganguli M. Burmeister L. A. Seaberg E. C. Belle S. DeKosky S. T. (1996). Association between dementia and elevated TSH: a community-based study. Biol. Psychiatry 40, 714–725. 10.1016/0006-3223(95)00489-0 8894063

[B14] Gulseren S. Gulseren L. Hekimsoy Z. Cetinay P. Ozen C. Tokatlioglu B. (2006). Depression, anxiety, health-related quality of life, and disability in patients with overt and subclinical thyroid dysfunction. Arch. Med. Res. 37, 133–139. 10.1016/j.arcmed.2005.05.008 16314199

[B15] Gussekloo J. van E. E. de Craen A. J. Meinders A. E. Frölich M. Westendorp R. G. (2004). Thyroid status, disability and cognitive function, and survival in old age. JAMA. 292, 2591–2599. 10.1001/jama.292.21.2591 15572717

[B16] Gussekloo J. van E. E. de Craen AJ Meinders, A. E. Frölich M. Westendorp R. G. (2006). [Thyroid function, activities of daily living and survival in extreme old age: the ‘Leiden 85-plus Study']. Ned. Tijdschr. Geneeskd. 150, 90–96. 16440564

[B17] Hedges L. V. Vevea J. L. (1998). Fixed-and random-effects models in meta-analysis. Psychol. Methods 3:486 10.1037/1082-989X.3.4.486

[B18] Helfand M. (2004). Screening for subclinical thyroid dysfunction in nonpregnant adults: a summary of the evidence for the U.S. preventive services task force. Ann. Intern. Med. 140, 128–141. 10.7326/0003-4819-140-2-200401200-00015 14734337

[B19] Hogervorst E. Huppert F. Matthews F. E. Brayne C. (2008). Thyroid function and cognitive decline in the MRC cognitive function and ageing study. Psychoneuroendocrinology 33, 1013–1022. 10.1016/j.psyneuen.2008.05.008 18640783

[B20] Huston P. Naylor C. D. (1996). Health services research: reporting on studies using secondary data sources. CMAJ 155, 1697–1709. 8976336 PMC1335495

[B21] Jensovsky J. Spackova N. Hejdukova B. Ruzicka E. (2000). [Effect of normalization of an isolated increase in TSH on the neuropsychological profile of patients]. Cas. Lek. Cesk. 139, 313–316. 10953421

[B22] Joffe R. T. Pearce E. N. Hennessey J. V. Ryan J. J. Stern R. A. (2013). Subclinical hypothyroidism, mood, and cognition in older adults: a review. Int. J. Geriatr. Psychiatry 28, 111–118. 10.1002/gps.3796 22410877 PMC3488161

[B23] St John J. A. Henderson V. W. Gatto N. M. McCleary C. A. Spencer C. A. Hodis H. N.. (2009). Mildly elevated TSH and cognition in middle-aged and older adults. Thyroid 19, 111–117. 10.1089/thy.2008.0226 19191743 PMC2715222

[B24] Jorde R. Waterloo K. Storhaug H. Nyrnes A. Sundsfjord J. Jenssen T. G. (2006). Neuropsychological function and symptoms in subjects with subclinical hypothyroidism and the effect of thyroxine treatment. J. Clin. Endocrinol. Metab. 91, 145–153. 10.1210/jc.2005-1775 16263815

[B25] Manciet G. Dartigues J. F. Decamps A. Barberger-Gateau P. Letenneur L. Latapie M. J.. (1995). The PAQUID survey and correlates of subclinical hypothyroidism in elderly community residents in the southwest of France. Age Ageing 24, 235–241. 10.1093/ageing/24.3.235 7645445

[B26] Park Y. J. Lee E. J. Lee Y. J. Choi S. H. Park J. H. Lee S. B.. (2010). Subclinical hypothyroidism (SCH) is not associated with metabolic derangement, cognitive impairment, depression or poor quality of life (QoL) in elderly subjects. Arch. Gerontol. Geriatr. 50, e68–e73. 10.1016/j.archger.2009.05.015 19545916

[B27] Parle J. Roberts L. Wilson S. Pattison H. Roalfe A. Haque M. S.. (2010). A randomized controlled trial of the effect of thyroxine replacement on cognitive function in community-living elderly subjects with subclinical hypothyroidism: the Birmingham elderly thyroid study. J. Clin. Endocrinol. Metab. 95, 3623–3632. 10.1210/jc.2009-2571 20501682

[B28] Parsaik A. K. Singh B. Roberts R. O. Pankratz S. Edwards K. K. Geda Y. E.. (2014). Hypothyroidism and risk of mild cognitive impairment in elderly persons: a population-based study. JAMA Neurol. 71, 201–207. 10.1001/jamaneurol.2013.5402 24378475 PMC4136444

[B29] Razvi S. Ingoe L. E. McMillan C. V. Weaver J. U. (2005). Health status in patients with sub-clinical hypothyroidism. Eur. J. Endocrinol. 152, 713–717. 10.1530/eje.1.01907 15879356

[B30] Resta F. Triggiani V. Barile G. Benigno M. Suppressa P. Giagulli V. A.. (2012). Subclinical hypothyroidism and cognitive dysfunction in the elderly. Endocr. Metab. Immune Disord. Drug Targets. 12, 260–267. 10.2174/187153012802002875 22385117

[B31] Roberts L. M. Pattison H. Roalfe A. Franklyn J. Wilson S. Hobbs F. D.. (2006). Is subclinical thyroid dysfunction in the elderly associated with depression or cognitive dysfunction? Ann. Intern. Med. 145, 573–581. 10.7326/0003-4819-145-8-200610170-00006 17043339

[B32] Rodondi N. den Elzen W. P. Bauer D. C. Cappola A. R. Razvi S. Walsh J. P.. (2010). Subclinical hypothyroidism and the risk of coronary heart disease and mortality. JAMA 304, 1365–1374. 10.1001/jama.2010.1361 20858880 PMC3923470

[B33] Samuels M. H. (2008). Cognitive function in untreated hypothyroidism and hyperthyroidism. Curr. Opin. Endocrinol. Diabetes Obes. 15, 429–433. 10.1097/MED.0b013e32830eb84c 18769215

[B34] Sawin C. T. Castelli W. P. Hershman J. M. McNamara P. Bacharach P. (1985). The aging thyroid. Thyroid deficiency in the Framingham study. Arch. Intern. Med. 145, 1386–1388. 10.1001/archinte.1985.00360080056006 4026469

[B35] Hulley S. B. Cummings S. R. Browner W. S. Grady D. Hearst N. Newman T. B. (2001). Designing Clinical Research: An Epidemiologic Approach. Philadelphia, PA: Lippincott Williams & Wilkins.

[B36] Stroup D. F. Berlin J. A. Morton S. C. Olkin I. Williamson G. D. Rennie D.. (2000). Meta-analysis of observational studies in epidemiology: a proposal for reporting. Meta-analysis Of Observational Studies in Epidemiology (MOOSE) group. JAMA 283, 2008–2012. 10.1001/jama.283.15.2008 10789670

[B37] Surks M. I. Ortiz E. Daniels G. H. Sawin C. T. Col N. F. Cobin R. H.. (2004). Subclinical thyroid disease: scientific review and guidelines for diagnosis and management. JAMA 291, 228–238. 10.1001/jama.291.2.228 14722150

[B38] Vanderpump M. P. Tunbridge W. M. French J. M. Appleton D. Bates D. Clark F.. (1995). The incidence of thyroid disorders in the community: a twenty-year follow-up of the Whickham Survey. Clin. Endocrinol. (Oxf). 43, 55–68. 10.1111/j.1365-2265.1995.tb01894.x 7641412

[B39] Vigário P. Teixeira P. Reuters V. Almeida C. Maia M. Silva M.. (2009). Perceived health status of women with overt and subclinical hypothyroidism. Med. Princ. Pract. 18, 317–322. 10.1159/000215731 19494541

[B40] Wiersinga W. M. (2006). [Uncertainties about the benefit of treatment in subclinical thyroid dysfunction]. Ned. Tijdschr. Geneeskd. 150, 71–74. 16440559

[B41] Wijsman L. W. de Craen A. J. Trompet S. Gussekloo J. Stott D. J. Rodondi N.. (2013). Subclinical thyroid dysfunction and cognitive decline in old age. PLoS ONE 8:e59199. 10.1371/journal.pone.0059199 23554996 PMC3595244

[B42] Yamamoto N. Ishizawa K. Ishikawa M. Yamanaka G. Yamanaka T. Murakami S.. (2012). Cognitive function with subclinical hypothyroidism in elderly people without dementia: one year follow up. Geriatr. Gerontol. Int. 12, 164–165. 10.1111/j.1447-0594.2011.00727.x 22188501

[B43] Zhu D. F. Wang Z. X. Zhang D. R. Pan Z. L. He S. Hu X. P.. (2006). fMRI revealed neural substrate for reversible working memory dysfunction in subclinical hypothyroidism. Brain 129(Pt 11), 2923–2930. 10.1093/brain/awl215 16921178

