# Supplementary material for: Subclinical hypothyroidism and cognitive function in people over 60 years: a systematic review and meta-analysis
Source: Front Aging Neurosci. 2015 Aug 11;7:150. doi: 10.3389/fnagi.2015.00150 (PMC4531303; doi:10.3389/fnagi.2015.00150)
Supplement: Supplementary file 1 [file DataSheet1.DOCX]

**Appendix 1**

Databases:

**PubMed**

<http://www.ncbi.nlm.nih.gov/entrez/query.fcgi?otool=leiden>

("subclinical hypothyroidism"[all fields] OR "subclinical hypothyroid"[all fields] OR "subclinical hypothyroids"[all fields] OR "sub-clinical hypothyroidism"[all fields] OR (("sub-clinical"[all fields] OR subclinical[all fields] OR subclin*[all fields] OR sub-clin*[all fields]) AND ("Hypothyroidism"[Mesh] OR hypothyroidism[all fields] OR hypothyroid[all fields] OR hypothyroids[all fields] OR hypothyroid*[all fields]))) AND ("Cognition"[mesh] OR "cognition"[All Fields] OR "Awareness"[mesh] OR "awareness"[All Fields] OR "Cognitive dissonance"[mesh] OR "cognitive dissonance"[All Fields] OR "Cognitive reserve"[mesh] OR "cognitive reserve"[All Fields] OR "Comprehension"[mesh] OR "comprehension"[all fields] OR "Consciousness"[mesh] OR "consciousness"[all fields] OR "Imagination"[mesh] OR "imagination"[all fields] OR "Dreams"[mesh] OR "dreams"[all fields] OR "Fantasy"[mesh] OR "fantasy"[all fields] OR "Intuition"[mesh] OR "intuition"[all fields] OR "Cognition disorders"[mesh] OR "cognitive"[all fields] OR "Mental Processes"[mesh] OR "anticipation"[all fields] OR "Imagination"[mesh] OR "imagination"[all fields] OR "Executive function"[mesh] OR "executive function"[all fields] OR "Higher nervous activity"[mesh] OR "higher nervous activity"[all fields] OR "Learning"[mesh] OR "learning"[all fields] OR "Avoidance learning"[mesh] OR "avoidance learning"[all fields] OR "Conditioning (psychology)"[mesh] OR "conditioning"[all fields] OR "Cues"[mesh] OR "cues"[all fields] OR "Inhibition (psychology)"[mesh] OR "inhibition"[all fields] OR "Memory"[mesh] OR "memory"[all fields] OR "memories"[all fields] OR "Neurolinguistic programming"[mesh] OR "neurolinguistic programming"[all fields] OR "Overlearning"[mesh] OR "overlearning"[all fields] OR "Problem solving"[mesh] OR "problem solving"[all fields] OR "Perception"[mesh] OR "perception"[all fields] OR "Discrimination (psychology)"[mesh] OR "discrimination"[all fields] OR "Field dependence-independence"[mesh] OR "field dependence-independence"[all fields] OR "field dependence independence"[all fields] OR "Illusions"[mesh] OR "illusions"[all fields] OR "illusion"[all fields] OR "pattern recognition"[all fields] OR "Perceptual distortion"[mesh] OR "perceptual distortion"[all fields] OR "Sensory deprivation"[mesh] OR "sensory deprivation"[all fields] OR "Sensory thresholds"[mesh] OR "sensory thresholds"[all fields] OR "sensory threshold"[all fields] OR "Sensation"[mesh] OR "sensation"[all fields] OR "Thinking"[mesh] OR "thinking"[all fields] OR "Concept formation"[mesh] OR "concept formation"[all fields] OR "Creativity"[mesh] OR "creativity"[all fields] OR "creativeness"[all fields] OR creative[all fields] OR "Decision making"[mesh] OR "decision making"[all fields] OR "Esthetics"[mesh] OR "esthetics"[all fields] OR "esthetic"[all fields] OR "aesthetics"[all fields] OR "aesthetic"[all fields] OR "Judgment"[mesh] OR "judgment"[all fields] OR "Volition"[mesh] OR "volition"[all fields] OR "Affect"[mesh] OR "affect"[all fields] OR "affective"[all fields] OR "mood"[all fields] OR "moods"[all fields] OR moody[all fields] OR "Emotions"[mesh] OR "emotions"[all fields] OR "emotion"[all fields] OR "emotional"[all fields] OR emotional*[all fields]) AND ("elderly"[all fields] OR elder[all fields] OR "Aged"[mesh] OR "older people"[all fields] OR "older patient"[all fields] OR "older patient"[all fields] OR "old patient"[all fields] OR "old patients"[all fields] OR "Aging"[mesh] OR "aging"[all fields] OR "ageing"[all fields] OR geriatr*[all fields] OR **"aged"[all fields] OR "old"[all fields] OR "oldest"[all fields] OR "older"[all fields]**)

**EMBASE**

<http://ovidsp.ovid.com/ovidweb.cgi?T=JS&PAGE=main&MODE=ovid&D=oemezd>

(("subclinical hypothyroidism" OR "subclinical hypothyroid" OR "subclinical hypothyroids" OR "sub-clinical hypothyroidism" OR "sub-clinical hypothyroid" OR "sub-clinical hypothyroids").af OR (("sub-clinical" OR subclinical OR subclin* OR sub-clin*).af AND (exp hypothyroidism/ OR hypothyroid*.af))) AND ((cognition OR Cognition OR Awareness OR Cognitive Dissonance OR Cognitive Reserve OR Comprehension OR Consciousness OR Imagination OR Dreams OR Fantasy OR Intuition OR Cognition Disorders OR cognitive OR Mental Processes OR Anticipation OR Imagination OR Executive Function OR Higher Nervous Activity OR Learning OR Avoidance Learning OR Conditioning OR Cues OR Inhibition OR Memory OR memories OR Neurolinguistic Programming OR Overlearning OR Problem Solving OR Perception OR Discrimination OR Field Dependence-Independence OR Illusions OR illusion OR Pattern Recognition OR Perceptual Distortion OR Sensory Deprivation OR Sensory Thresholds OR sensation OR Thinking OR Concept Formation OR Creativeness OR creative OR Decision Making OR Esthetics OR esthetic OR aesthetics OR aesthetic OR Judgment OR Volition OR mood OR moods OR moody OR emotion OR emotions OR emotional* OR affect OR affective).af OR exp Cognition/ OR exp mental function/) AND (exp aged/ OR elder*.af OR "older people".af OR exp aging/ OR aging.af OR ageing.af OR "older people".af OR "older patient".af OR "older patient".af OR "old patient".af OR "old patients".af OR "aging".af OR "ageing".af OR geriatr*.af OR **"aged".ti,ab OR "old".ti,ab OR "oldest".ti,ab OR "older".ti,ab**)

**Web of Science**

<http://isiknowledge.com/wos>

TS=(("sub-clinical" OR subclinical OR subclin* OR "sub-clin*") AND (hypothyroidism OR hypothyroid OR hypothyroids OR hypothyroid*)) AND TS=("elderly" OR elder* OR "older people" OR "aging" OR "ageing" OR "aged" OR "old" OR "older" OR "oldest") AND TS=(cognit* OR cognition OR Cognition OR Awareness OR "Cognitive Dissonance" OR "Cognitive Reserve" OR Comprehension OR Consciousness OR Imagination OR Dreams OR Fantasy OR Intuition OR "Cognition Disorders" OR cognitive OR "Mental Proces*" OR Anticipation OR Imagination OR "Executive Function" OR "Higher Nervous Activity" OR Learning OR "Avoidance Learning" OR Conditioning OR Cues OR Inhibition OR Memory OR memories OR "Neurolinguistic Programming" OR Overlearning OR "Problem Solving" OR perception OR Discrimination OR "Field Dependence-Independence" OR Illusions OR illusion OR "Pattern Recognition" OR "Perceptual Distortion" OR "Sensory Deprivation" OR "Sensory Thresholds" OR sensation OR Thinking OR "Concept Formation" OR Creativeness OR creative OR "Decision Making" OR Esthetics OR esthetic OR aesthetics OR aesthetic OR Judgment OR Volition OR mood OR moods OR moody OR emotion OR emotions OR emotional* OR affect OR affective)

**Cochrane**

<http://www.thecochranelibrary.com/view/0/index.html>

(("sub-clinical" OR subclinical OR subclin* OR sub-clin*) AND (hypothyroidism OR hypothyroid OR hypothyroids OR hypothyroid*)) AND (aged OR elderly OR elder* OR "older people" OR aging OR ageing OR old OR older OR oldest) AND (cognit* OR cognition OR Cognition OR Awareness OR Cognitive Dissonance OR Cognitive Reserve OR Comprehension OR Consciousness OR Imagination OR Dreams OR Fantasy OR Intuition OR Cognition Disorders OR cognitive OR "Mental Processes" OR Anticipation OR Imagination OR Executive Function OR Higher Nervous Activity OR Learning OR Avoidance Learning OR Conditioning OR Cues OR Inhibition OR Memory OR memories OR Neurolinguistic Programming OR Overlearning OR Problem Solving OR perception OR Discrimination OR Field Dependence-Independence OR Illusions OR illusion OR Pattern Recognition OR Perceptual Distortion OR Sensory Deprivation OR Sensory Thresholds OR sensation OR Thinking OR Concept Formation OR Creativeness OR creative OR Decision Making OR Esthetics OR esthetic OR aesthetics OR aesthetic OR Judgment OR Volition OR mood OR moods OR moody OR emotion OR emotions OR emotional* OR affect OR affective)

**CENTRAL**

<http://crso.cochrane.org/>

schoones/

(("sub-clinical" OR subclinical OR subclin* OR sub-clin*) AND (hypothyroidism OR hypothyroid OR hypothyroids OR hypothyroid*)) AND (aged OR elderly OR elder* OR "older people" OR aging OR ageing OR old OR older OR oldest) AND (cognit* OR cognition OR Cognition OR Awareness OR Cognitive Dissonance OR Cognitive Reserve OR Comprehension OR Consciousness OR Imagination OR Dreams OR Fantasy OR Intuition OR Cognition Disorders OR cognitive OR "Mental Processes" OR Anticipation OR Imagination OR Executive Function OR Higher Nervous Activity OR Learning OR Avoidance Learning OR Conditioning OR Cues OR Inhibition OR Memory OR memories OR Neurolinguistic Programming OR Overlearning OR Problem Solving OR perception OR Discrimination OR Field Dependence-Independence OR Illusions OR illusion OR Pattern Recognition OR Perceptual Distortion OR Sensory Deprivation OR Sensory Thresholds OR sensation OR Thinking OR Concept Formation OR Creativeness OR creative OR Decision Making OR Esthetics OR esthetic OR aesthetics OR aesthetic OR Judgment OR Volition OR mood OR moods OR moody OR emotion OR emotions OR emotional* OR affect OR affective)

**CINAHL**

<http://search.ebscohost.com/login.aspx?authtype=ip,uid&profile=lumc&defaultdb=cin20>

(("sub-clinical" OR subclinical OR subclin* OR sub-clin*) AND (hypothyroidism OR hypothyroid OR hypothyroids OR hypothyroid*)) AND (aged OR elderly OR elder* OR "older people" OR aging OR ageing OR old OR older OR oldest) AND (cognit* OR cognition OR Cognition OR Awareness OR Cognitive Dissonance OR Cognitive Reserve OR Comprehension OR Consciousness OR Imagination OR Dreams OR Fantasy OR Intuition OR Cognition Disorders OR cognitive OR "Mental Processes" OR Anticipation OR Imagination OR Executive Function OR Higher Nervous Activity OR Learning OR Avoidance Learning OR Conditioning OR Cues OR Inhibition OR Memory OR memories OR Neurolinguistic Programming OR Overlearning OR Problem Solving OR perception OR Discrimination OR Field Dependence-Independence OR Illusions OR illusion OR Pattern Recognition OR Perceptual Distortion OR Sensory Deprivation OR Sensory Thresholds OR sensation OR Thinking OR Concept Formation OR Creativeness OR creative OR Decision Making OR Esthetics OR esthetic OR aesthetics OR aesthetic OR Judgment OR Volition OR mood OR moods OR moody OR emotion OR emotions OR emotional* OR affect OR affective)

**PsycINFO**

<http://search.ebscohost.com/login.aspx?authtype=ip,uid&profile=lumc&defaultdb=psyh>

(("sub-clinical" OR subclinical OR subclin* OR sub-clin*) AND (hypothyroidism OR hypothyroid OR hypothyroids OR hypothyroid*)) AND (aged OR elderly OR elder* OR "older people" OR aging OR ageing OR old OR older OR oldest) AND (cognit* OR cognition OR Cognition OR Awareness OR Cognitive Dissonance OR Cognitive Reserve OR Comprehension OR Consciousness OR Imagination OR Dreams OR Fantasy OR Intuition OR Cognition Disorders OR cognitive OR "Mental Processes" OR Anticipation OR Imagination OR Executive Function OR Higher Nervous Activity OR Learning OR Avoidance Learning OR Conditioning OR Cues OR Inhibition OR Memory OR memories OR Neurolinguistic Programming OR Overlearning OR Problem Solving OR perception OR Discrimination OR Field Dependence-Independence OR Illusions OR illusion OR Pattern Recognition OR Perceptual Distortion OR Sensory Deprivation OR Sensory Thresholds OR sensation OR Thinking OR Concept Formation OR Creativeness OR creative OR Decision Making OR Esthetics OR esthetic OR aesthetics OR aesthetic OR Judgment OR Volition OR mood OR moods OR moody OR emotion OR emotions OR emotional* OR affect OR affective)

**Academic Search Premier [fulltextzoeken]**

<http://digitallibrary.leidenuniv.nl/V?func=native-link&resource=LDN00184>

TITLE

ABSTRACT

(("sub-clinical" OR subclinical OR subclin* OR sub-clin*) AND (hypothyroidism OR hypothyroid OR hypothyroids OR hypothyroid*)) AND (aged OR elderly OR elder* OR "older people" OR aging OR ageing OR old OR older OR oldest) AND (cognit* OR cognition OR Cognition OR Awareness OR Cognitive Dissonance OR Cognitive Reserve OR Comprehension OR Consciousness OR Imagination OR Dreams OR Fantasy OR Intuition OR Cognition Disorders OR cognitive OR "Mental Processes" OR Anticipation OR Imagination OR Executive Function OR Higher Nervous Activity OR Learning OR Avoidance Learning OR Conditioning OR Cues OR Inhibition OR Memory OR memories OR Neurolinguistic Programming OR Overlearning OR Problem Solving OR perception OR Discrimination OR Field Dependence-Independence OR Illusions OR illusion OR Pattern Recognition OR Perceptual Distortion OR Sensory Deprivation OR Sensory Thresholds OR sensation OR Thinking OR Concept Formation OR Creativeness OR creative OR Decision Making OR Esthetics OR esthetic OR aesthetics OR aesthetic OR Judgment OR Volition OR mood OR moods OR moody OR emotion OR emotions OR emotional* OR affect OR affective)

("subclinical hypothyroidism" OR "subclinical hypothyroid" OR "subclinical hypothyroids" OR "sub-clinical hypothyroidism" OR "sub-clinical hypothyroid" OR "sub-clinical hypothyroids") AND (aged OR elderly OR elder* OR "older people" OR aging OR ageing OR old OR older OR oldest) AND (cognit* OR cognition OR Cognition OR Awareness OR Cognitive Dissonance OR Cognitive Reserve OR Comprehension OR Consciousness OR Imagination OR Dreams OR Fantasy OR Intuition OR Cognition Disorders OR cognitive OR "Mental Processes" OR Anticipation OR Imagination OR Executive Function OR Higher Nervous Activity OR Learning OR Avoidance Learning OR Conditioning OR Cues OR Inhibition OR Memory OR memories OR Neurolinguistic Programming OR Overlearning OR Problem Solving OR perception OR Discrimination OR Field Dependence-Independence OR Illusions OR illusion OR Pattern Recognition OR Perceptual Distortion OR Sensory Deprivation OR Sensory Thresholds OR sensation OR Thinking OR Concept Formation OR Creativeness OR creative OR Decision Making OR Esthetics OR esthetic OR aesthetics OR aesthetic OR Judgment OR Volition OR mood OR moods OR moody OR emotion OR emotions OR emotional* OR affect OR affective)
