# Supplementary material for: Subclinical hypothyroidism and cognitive function in people over 60 years: a systematic review and meta-analysis
Source: Front Aging Neurosci. 2015 Aug 11;7:150. doi: 10.3389/fnagi.2015.00150 (PMC4531303; doi:10.3389/fnagi.2015.00150)
Supplement: Supplementary file 2 [file DataSheet2.DOCX]

**Appendix 2:**

Quality assessment of included studies

|  | **First author** | **Clear hypothesis** | **Population** | **SCH properly defined** | **Detailed methods description** | **Validity of measures** | **Cognitive domains tested** | **Statistical methods** | **Adjustments** | **Clear presentation of results** | **Generalizability** | **Formal adjudication procedure** | **Overall score** |
| --- | --- | --- | --- | --- | --- | --- | --- | --- | --- | --- | --- | --- | --- |
| 1 | Roberts et al, 2006 | 2 | 2 | 2 | 2 | 2 | 1 | 2 | 2 | 2 | 2 | 2 | 21 |
| 2 | Wijsman et al, 2013 | 2 | 2 | 2 | 2 | 2 | 2 | 2 | 2 | 2 | 2 | 2 | 22 |
| 3 | Parsaik et al, 2014 | 2 | 2 | 2 | 2 | 2 | 1 | 2 | 2 | 2 | 2 | 2 | 21 |
| 4 | Park et al, 2010 | 2 | 2 | 2 | 2 | 2 | 2 | 2 | 1 | 2 | 1 | 1 | 19 |
| 5 | De Jongh et al, 2011 | 2 | 2 | 2 | 2 | 2 | 2 | 2 | 1 | 1 | 2 | 2 | 20 |
| 6 | Hogervorst et al, 2008 | 2 | 2 | 2 | 2 | 2 | 1 | 2 | 2 | 1 | 2 | 2 | 20 |
| 7 | Gussekloo et al*,* 2004 | 2 | 2 | 2 | 2 | 2 | 2 | 2 | 2 | 1 | 2 | 1 | 21* |
| 8 | John et al, 2009 | 2 | 1 | 1 | 2 | 2 | 2 | 2 | 2 | 1 | 1 | 1 | 17 |
| 9 | Resta et al, 2012 | 2 | 1 | 1 | 2 | 2 | 2 | 1 | 2 | 1 | 1 | 1 | 16 |
| 10 | Ceresini et al, 2009 | 2 | 2 | 2 | 1 | 1 | 1 | 2 | 2 | 2 | 1 | 2 | 18 |
| 11 | Formiga et al, 2014 | 2 | 2 | 2 | 2 | 2 | 1 | 1 | 1 | 2 | 2 | 2 | 19* |
| 12 | Manciet et al, 1995 | 2 | 2 | 2 | 2 | 2 | 2 | 1 | - | 1 | 1 | 1 | 16 |
| 13 | Yamamoto et al, 2012 | 2 | 2 | 1 | 1 | 2 | 1 | 1 | - | 2 | 2 | 2 | 16 |
| 14 | Cook et al, 2002 | 2 | 1 | 1 | 2 | 2 | 2 | 1 | 2 | 1 | 1 | 1 | 16 |
| 15 | Cardenas- Ibarra et al, 2008 | 2 | 1 | 1 | 1 | 1 | 1 | 1 | 1 | 1 | 1 | 1 | 12 |

*Score based on published and unpublished data provided by the author
